# The Value of Statistics and Evidence-Based Medicine in the Care of Neurosurgical Patients

**DOI:** 10.7759/cureus.27455

**Published:** 2022-07-29

**Authors:** Aizik Wolf, Michael W McDermott, Mark N Hadley

**Affiliations:** 1 Neurosurgery, Miami Neuroscience Center at Larkin, Miami, USA; 2 Neurological Surgery, University of California, San Francisco, USA; 3 Neurological Surgery, University of Alabama, Birmingham, USA

**Keywords:** neurosurgical, medicine, based, evidence, statistics

## Abstract

Science and the art of surgery should be anchored on evidence-based medicine. There is no room in the discipline of neurosurgery for “personal anecdotes/experience,” and the concept of “hero worship.” The construction of evidence-based medicine guidelines is essential in our continued improvement of care for neurosurgical patients.

## Editorial

At the recent 17th World Congress of Neurosurgery in Bogotá, Colombia, held March 13-18, 2022, a prominent neurosurgeon stated, while moderating the talk I was giving on The Treatment of Large Acoustic Schwannomas with Gamma Knife Radiosurgery, that "statistics are not useful in neurosurgery". The reaction of the audience in attendance was one that made me uncomfortable. Certainly, for younger neurosurgeons or those in training, this kind of statement requires comment.

A brief survey of publications in neurosurgery combining the terms "statistics AND neurosurgery" reveals 3,348 citations, and with no time limits, 35,680 citations. Obviously, statistics and the art and science (evidence-based medicine) of neurosurgery are not only relevant but essential. The disciplined science of evidence-based medicine applied to neurosurgery allows us to distinguish between the most efficacious management strategies including the diagnosis, assessment, and treatment of patients with neurosurgical diseases based on the academic rigor of studies in our literature on these topics. The compilation of evidenced-based medicine in topic-specific referred guidelines provides a chronicle of accepted strategies for assessment, diagnosis, treatment, and prognosis, ranking them according to scientific merit. Properly prepared and constructed evidence-based medicine guidelines allow practitioners to appreciate the variety of acceptable strategies they might employ for a given patient with a given pathology and identifies an area where little to no sound evidence exists, driving further research to fill these voids.

Organized neurosurgery has done much in the past 25 years to use scientific evidence-derived data to guide clinical care. Our practitioners and neurosurgical societies have published over 250 evidence-based guidelines for virtually every neurosurgical subspecialty area in our profession. Class I medical evidence is derived from randomized, blinded clinical trials, properly designed, carried out, and completed. Class II medical evidence (less scientifically robust but still substantial) is derived from properly designed and completed comparative studies or case-control studies. Class III medical evidence is derived from case series or poorly designed or carried out (flawed) attempts at Class I or Class II trials. Class IV medical evidence is derived from historical "the way I do it" assessment, diagnosis, or treatment proclamations. This may have been what the acclaimed neurosurgeon was speaking about in his dissertation in Bogota. While statistical facts apply to groups and populations of similar patients with similar pathology, statistics don't always apply to an individual patient due to other medical conditions, allergies, or pathology that an individual may have. Nonetheless, statistics and knowledge of evidence-based medical facts remain critically important in the contemporary, comprehensive care of our patients.

Much of how we currently practice is based on Class III medical evidence, but year by year new studies of these issues bring important Class II medical evidence, sometimes Class I medical evidence to light. As more and more practitioners and residents in training learn the principles of evidence-based medicine and apply them to design studies to answer clinical questions, our field advances. At the institutional level, internal analysis of case series (Class III medical evidence), while not as robust scientifically as evidence derived from randomized trials or properly designed comparative studies, can provide meaningful evidence on the incidence of a variety of different strategies to a similar pathology which can be used to help define the risk-benefit ratio and potential success of medical treatment or surgical intervention before that strategy is implemented. It can identify practitioners who perform less well or have inferior results compared to their peers allowing for physician education, training, and improvement. This kind of medical evidence can be useful when properly applied.

The decision to intervene for a patient with a newly diagnosed condition is based on a variety of patient factors such as age, associated medical conditions, frailty indices, performance status, and patient desires, in addition to the specific nature of that patient’s neurosurgical pathology. Certainly, all of these factors should be considered and patient treatment decisions must indeed be individualized. Knowledge of the existing evidence-based medical science about the specific neurosurgical pathology to be treated expands the expertise of the neurosurgical practitioner and allows the treating surgeon to better stylize and individualize patient care. 

An example of how statistics and evidence-based medicine are helpful in guiding decisions for treatment is the recent international study on tuberculum sellae meningiomas, which in most institutional surgical series are rare tumors accounting for only 2% to 4% of all cases. The recruitment and collaborative collation of data from many institutions treating these tumors provide increased statistical power for the decision-making that guides the surgical approaches and treatment for patients with this pathology. An evidence-based statistical analysis suggests that for smaller tumors, an endoscopic endonasal approach for surgical removal provides better long-term tumor control than historical or individual surgeon preferences and practices.

There are many excellent examples where statistical evidence-based medicine analyses of the literature provide the basis for guideline recommendations based on scientific merit for diagnosis, assessment, treatment, and prognosis in neurosurgery. Spinal neurosurgery is the most common form of neurosurgical intervention in contemporary neurosurgery practice. Evidence-based guidelines have been developed for Lumbar Spinal Fusion procedures on 20 topics related to lumbar spinal pathology. The Guidelines for the Management of Acute Traumatic Cervical Spine and Spinal Cord Injuries and the Guidelines for the Management of Cervical Myelopathy are two other comprehensive and contemporary evidence-based compendia on the diagnosis, assessment, management, and prognosis of these disorders that guide patient care based on existing scientific knowledge and "statistics". These focused, topic-specific guidelines and many others in neurosurgery have been carefully produced, vetted, refereed, and have been approved/adopted by our governing specialty societies, the American Association of Neurological Surgeons, and the Congress of Neurological Surgeons as official doctrine [1-3].

We do not argue that individual patient decision-making is a complex process and must be individualized. However, the broad statement that "statistics are not useful in neurosurgery" grossly misrepresents 25 years of concerted effort to apply the science of evidence-based medicine to disease states and pathology within our specialty to improve the care offered by neurosurgeons and the outcomes of the patients we treat. There is no room in neurosurgery for "hero worshiping" and blind trust. We need to always validate our "feelings" and "memory."
